# Exploring the Inhibition of *Toxoplasma gondii* α-Carbonic Anhydrase by Sulfonamides: Insights into Potential Drug Targeting

**DOI:** 10.3390/ijms26010116

**Published:** 2024-12-26

**Authors:** Simone Giovannuzzi, Viviana De Luca, Clemente Capasso, Claudiu T. Supuran

**Affiliations:** 1 Neurofarba Department, Section of Pharmaceutical Sciences, University of Florence, Via Ugo Schiff 6, Sesto Fiorentino, 50019 Florence, Italy; simone.giovannuzzi@unifi.it (S.G.); claudiu.supuran@unifi.it (C.T.S.); 2 Department of Biology, Agriculture and Food Sciences, National Research Council (CNR), Institute of Biosciences and Bioresources, 80131 Naples, Italy; viviana.deluca@ibbr.cnr.it

**Keywords:** carbonic anhydrase, inhibition constants, protozoan parasite, sulfonamide-based inhibitors, *Toxoplasma gondii*, toxoplasmosis

## Abstract

*Toxoplasma gondii*, the causative agent of toxoplasmosis, is a protozoan parasite capable of infecting a wide range of hosts, posing significant health risks, particularly to immunocompromised individuals and congenital transmission. Current therapeutic options primarily target the active tachyzoite stage but are limited by issues such as toxicity and incomplete efficacy. As a result, there is an urgent need for alternative therapies that can selectively target parasite-specific mechanisms critical for metabolic processes and host–parasite interactions. In this context, α-carbonic anhydrase (Tg_CA), an enzyme essential for *T. gondii* survival has emerged as a promising drug target. Tg_CA was successfully expressed and purified to evaluate its susceptibility to sulfonamide-based inhibitors, represented by compounds **1**–**24** and the **AAZ**–**HCT** series. These inhibitors demonstrated a broad spectrum of activity, with KI values ranging from 17.8 to 8450 nM. Several compounds exhibited moderate to high potency against Tg_CA; however, concerns regarding selectivity arose because of the inhibition of human isoforms, particularly CA I and CA II. Thus, although some inhibitors showed strong activity against Tg_CA, optimizing selectivity remains crucial for minimizing off-target effects and improving therapeutic efficacy. Further structural modifications may enhance selectivity and advance the development of effective treatments for toxoplasmosis.

## 1. Introduction

*Toxoplasma gondii*, a protozoan parasite belonging to the phylum Apicomplexa, is the etiological agent of toxoplasmosis, a globally prevalent disease with a broad spectrum of clinical manifestations [1,2]. Toxoplasmosis is classified as a zoonosis, meaning it can be transmitted from animals to humans, affecting a diverse range of hosts, including mammals and birds [3,4]. The parasite has multiple transmission routes, including ingestion of oocysts shed by infected cats, consumption of undercooked meat containing tissue cysts, and vertical transmission from an infected mother to her fetus during pregnancy [5,6,7]. While infections in immunocompetent individuals are often asymptomatic or mild, severe complications can arise in immunocompromised individuals and during congenital transmission, potentially leading to encephalitis, chorioretinitis, or systemic infections [5,6,7]. Beyond its impact on human health, the zoonotic nature of *T. gondii* also presents significant challenges to animal health, contributing to reproductive issues in livestock and affecting wildlife populations [4]. Thus, *T. gondii* remains a major concern in both clinical and veterinary settings [4,8]. Current therapeutic options, such as the antifolate drugs pyrimethamine (PYR) and trimethoprim (TMP) with sulfonamides, such as sulfadiazine (SDZ) and sulfamethoxazole (SMZ) [9], primarily target active tachyzoites but are limited by toxicity, poor patient tolerance, and incomplete efficacy against latent tissue cysts [10,11,12,13,14]. Spiramycin, a macrolide antibiotic, is another key drug used primarily to prevent maternal-fetal transmission of *T. gondii* during pregnancy [15,16,17]. However, owing to limited placental transfer, it is ineffective in treating established fetal infections. These limitations highlight the pressing need for alternative therapeutic strategies capable of targeting both the active tachyzoite and dormant bradyzoite stages of *T. gondii* infection. Current research focuses on two main areas: exploring alternative drug combinations, such as PYR with clindamycin or azithromycin [18], and identifying novel molecular targets, including the electron transport chain [19], fatty acid synthesis [20], isoprenoid pathway [21], gene expression control, cAMP signaling pathways [22], and calcium-dependent pathways [23]. In the pursuit of therapies targeting parasite-specific pathways critical for metabolic functions and host–parasite interactions, *T. gondii* α-carbonic anhydrase (Tg_CA) has emerged as a promising candidate [24]. This metalloenzyme, encoded by the parasite genome, plays a crucial role in maintaining metabolic homeostasis and could serve as a valuable target for novel therapeutic interventions [24]. Carbonic anhydrases (CAs) catalyze the reversible hydration of carbon dioxide to bicarbonate and protons (CO_2_ + H_2_O ⇌ HCO_3_^−^ + H^+^), a reaction critical for pH regulation, ion transport, and metabolic homeostasis [14,24,25,26]. In *T. gondii*, the parasitophorous vacuole (PV) is a specialized compartment that supports parasite survival and replication within the host cells. Evidence suggests that the PV environment plays a pivotal role in maintaining the intracellular pH balance, which is vital for parasite survival [27,28,29]. Changes in pH within the PV have been linked to important parasitic processes, such as egress and microneme secretion. The PV becomes acidified during the late stages of infection, facilitating critical activities such as the activation of pore-forming proteins like PLP1, which aid the parasite from escaping from host cells [30]. By modulating bicarbonate and proton levels, Tg_CA may play a key role in maintaining an optimal intracellular environment within the acidic confines of the PV, thereby supporting metabolic adaptability and resilience of the parasite [24]. In addition to pH regulation, Tg_CA may be involved in several key metabolic pathways essential for *T. gondii* survival, including gluconeogenesis, urea synthesis, and lipid metabolism [14,24,26]. These processes not only provide energy and structural components but also facilitate host–parasite interactions. In particular, lipids serve as energy reservoirs, cellular scaffolds, and signaling molecules that help modulate the host environment [31]. Furthermore, the identification of carbonic anhydrase-related proteins (CARPs), such as TgCA_RP, in *T. gondii* highlights the importance of CAs. TgCA_RP, a GPI-anchored protein, has been implicated in rhoptry biogenesis, a process crucial for host cell invasion and parasite survival [32]. Studies on α-CAs from other protozoan parasites, such as *Trypanosoma cruzi*, have demonstrated susceptibility to a range of CA inhibitors (CAIs), including sulfonamides, thiols, and hydroxamates [33,34,35]. These findings suggest that α-CA from *T. gondii* is a viable drug target [25]. Sulfonamides, a well-established class of CAIs, inhibit enzyme activity by coordinating with the zinc ion at the CA active site via their sulfonamide group [36,37,38]. Although their efficacy against human and bacterial CAs is well documented, their potential to inhibit protozoan α-CAs, including those from *T. gondii*, has been less thoroughly explored. Understanding the inhibition profiles of sulfonamide derivatives against *T. gondii* α-CAs may provide valuable insights for the development of selective and effective antiprotozoal agents [25]. Addressing these challenges is crucial for overcoming the current therapeutic limitations and advancing the development of safer and more effective treatments for toxoplasmosis [39].

In this context, the in vitro sulfonamide inhibition profile of recombinant *Toxoplasma gondii* α-carbonic anhydrase (Tg_CA) was investigated and compared with the inhibition patterns of two human CA isoforms (hCA I and hCA II). The potential selectivity of these inhibitors was evaluated by examining whether they could specifically target Tg_CA without significantly affecting human CAs, thus offering a therapeutic advantage by minimizing the off-target effects on human enzymes. By elucidating the distinct inhibition profiles of Tg_CA, this work contributes to the development of safer and more effective antiprotozoal therapies. Furthermore, it establishes a framework for selectively targeting pathogen-specific CAs in other infectious diseases, paving the way for innovative drug discovery and therapeutic strategies.

## 2. Results and Discussion

### 2.1. Catalytic Features of Tg_CA: Implications for Inhibitor Development

**Figure 1:**
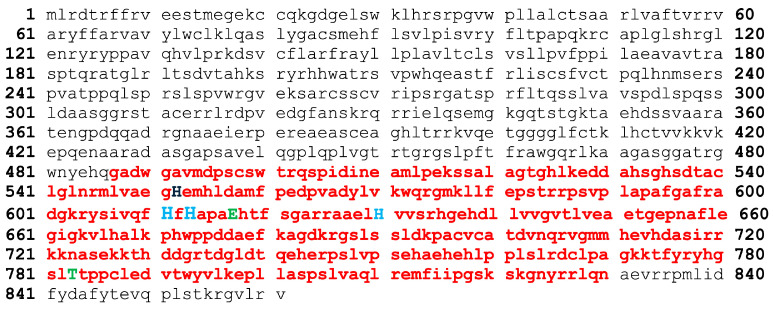
Amino acid sequence of *T. gondii* α-CA domain. This sequence highlights key functional regions and conserved residues. The α-CA domain is shown in red lowercase letters, the proton shuttle residue in black uppercase letter, the histidines involved in Zn^2+^ coordination in light blue uppercase letters, and the residues regulating access to the active site in green uppercase letters. These features emphasize the structural and functional significance of the enzyme active site and its potential catalytic activity.

In our previous work [24], we identified and characterized a homolog of human carbonic anhydrase in the *Toxoplasma gondii* genome. Building upon these foundational findings, the current study investigates the mechanisms underlying Tg_CA catalysis and their implications for inhibitor development. This focus provides novel insights that are crucial for targeting this enzyme therapeutically. To account for the genetic diversity among archetypal strains of *T. gondii*, a BLASTP search was performed across multiple *T. gondii* genomes using human α-CA I and II as reference sequences. This analysis identified a homologous α-CA encoded by *TGME49_259950* in the genome of the archetypal strain *T. gondii* ME49. The gene encodes a protein of 861 amino acid residues, which was retrieved from NCBI with ID XP_002365178.2, and is referred to as Tg_CA. Functional annotation described this protein as a “carbonate dehydratase, eukaryotic-type domain-containing protein”, indicating the presence of a structurally and functionally significant α-CA domain embedded within its larger sequence (Figure 1). Additionally, homologous α-CAs have been identified in other *T. gondii* strains, highlighting the evolutionary conservation of this domain across different *T. gondii* genomes [24].

Bioinformatic analysis confirmed the presence of conserved motifs and residues characteristic of α-CAs, including those involved in Zn^2+^ coordination and proton transfer, which are essential for enzymatic activity. The conserved residues observed in Tg_CA aligned closely with those found in other α-CAs, underscoring its potential role in maintaining CO_2_/HCO_3_^−^ homeostasis, as detailed in our previous study [24]. A similarity search of Tg_CA against a database of amino acid sequences (NCBI non-redundant protein database, nr) revealed several significant hits with α-CAs from different organisms (Table 1). The hits represent sequences with high similarity to *T. gondii* ME49 α-CA, demonstrating the strong evolutionary conservation of this domain and confirming its functional relevance in the identified protein.

The strong primary sequence similarity score (E-value) observed in Table 1 supports the hypothesis that α-CA identified in *T. gondii* shares structural and functional characteristics with its prokaryotic and eukaryotic counterparts. However, it is likely that adaptation of Tg_CA to a parasitic lifestyle introduces subtle amino acid variations, potentially fine-tuning its function to meet the unique metabolic demands of *T. gondii*. These variations could play a crucial role in optimizing the enzyme activity under parasitic conditions.

### 2.2. Recombinant Production and Purification of Tg_CA

**Figure 2:**
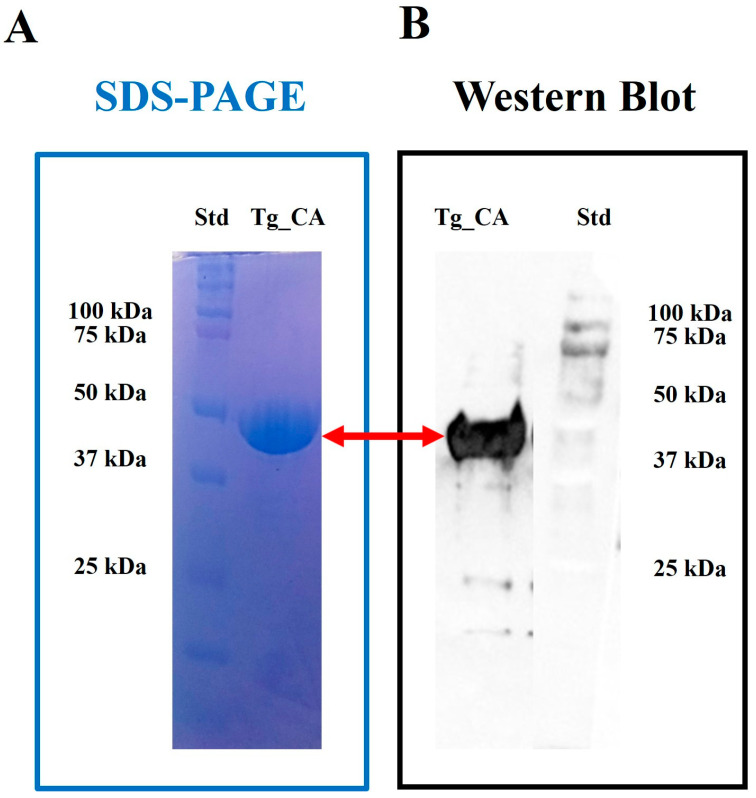
SDS-PAGE and Western blot analyses were used to assess the recombinant Tg_CA protein, purified using an affinity column. In SDS-PAGE (Panel (**A**)), the overexpressed recombinant protein in Lane Tg_CA was clearly visible by Coomassie staining. In the Western blot (Panel (**B**)), the protein in Lane Tg_CA was detected using an anti-His-tag antibody. Lane STD shows molecular markers (from bottom to top: 25, 37, 50, 75, and 100 kDa). Both techniques confirmed that the recombinant Tg_CA fusion protein had the expected molecular weight (47 kDa).

Given the strong conservation of the α-CA domain in Tg_CA and its critical role in CO_2_/HCO_3_^−^ homeostasis, we decided to produce a recombinant Tg_CA protein for further investigation. Recombinant protein production allows us to obtain enough enzyme to conduct detailed biochemical assays and study its enzymatic properties in a controlled setting. Thus, the gene encoding Tg_CA (from 487 to 830, Figure 1) was fused with hexahistidine (His-Tag) at the N-terminal end, which not only facilitates protein purification but also enables precise tracking of the enzyme during purification. The protein was heterologously expressed in *Escherichia coli*. Given the critical role of zinc in enzyme catalytic activity, we supplemented the culture medium with ZnCl_2_ (0.5 mM) to promote proper protein folding and ensure that the active site was correctly assembled. After lysis of bacterial cells by sonication and subsequent centrifugation to remove insoluble material, a significant portion of the recombinant protein was recovered in the soluble fraction. The protein was purified by nickel affinity chromatography using a His-Select HF Nickel column. SDS-PAGE analysis confirmed the homogeneity of the recombinant enzyme, showing a major band of approximately 47 kDa corresponding to the Tg_CA fusion protein (Figure 2A). The purification process yielded 5 mg/mL of Tg_CA protein, with a purity greater than 95%, as demonstrated by polyacrylamide gel electrophoresis (Figure 2A).

The molecular weight of the purified Tg_CA protein corresponded closely with its predicted size, further confirming the accuracy of the gene construct and expression system. This result reflects the fidelity of the heterologous expression process in *E. coli* as well as the stability of the recombinant protein during expression and purification. Achieving such homogeneity is critical for ensuring reproducibility in kinetic assays and inhibition screening. Moreover, the ability to produce large quantities of pure Tg_CA opens the possibility for future structural analyses, which could provide insights into the active site architecture of the enzyme. In addition, the enzymatic activity of the purified recombinant protein was demonstrated by protonography, as reported in our earlier study [24]. To further confirm the successful overexpression and identity of the recombinant protein, we performed a Western blot analysis using an anti-His antibody. This analysis provided strong evidence of successful overexpression, confirming the presence of Tg_CA protein in the soluble fraction of the bacterial extract (Figure 2B). Clear detection of a band at the expected molecular weight further validated the identity of the recombinant protein and the effectiveness of the purification process. Additionally, two recombinant human isoforms, CA I and CA II, were also produced in our lab with a purity greater than 90%, serving as benchmarks for comparison in our studies.

### 2.3. Kinetics of Tg_CA and Human CA Isoforms

The kinetic parameters of Tg_CA, hCA I, and hCA II were determined using a stopped-flow technique coupled with a pH-sensitive dye. This method enables rapid measurement of enzyme activity by monitoring pH changes as the reaction progresses. During CO_2_ hydration, the enzyme catalyzes the conversion of CO_2_ to bicarbonate, resulting in a shift in pH. A pH-sensitive dye was used to track these changes, providing real-time data on the catalytic activity of the enzyme. The stopped-flow technique, combined with the dye, allows for the precise monitoring of rapid enzymatic reactions by mixing the enzyme with its substrate (CO_2_) and observing the resulting pH shift over time. The kinetic analysis revealed that Tg_CA catalyzes the hydration of CO_2_ to bicarbonate with moderate efficiency, exhibiting a turnover number (kcat) of 2.16 × 10^5^ s^−1^ and a Michaelis constant (K_M_) of 16.0 mM. When compared to human carbonic anhydrases, Tg_CA shows a lower kcat, approximately ten times less than hCA II (1.40 × 10^6^), which is known for its highly efficient CO_2_ hydration. Compared to hCA I, Tg_CA exhibits a similar k_cat_ value of around 2.00 × 10^5^ s^−1^, which is of the same order of magnitude. In a previous manuscript, we constructed a model of the *T. gondii* carbonic anhydrase (Tg_CA) based on sequence alignment and structural data [24]. This analysis revealed key differences from human enzymes, including the presence of unique insertions in Tg_CA. These insertions are formed by loops of 31 and 82 amino acids, absent in hCA II [24]. From this model, it is difficult to fully understand how the structural variations, particularly the unique loops and insertions in Tg_CA, impact its enzymatic function. Although the model provides valuable insights into the overall structure of the enzyme, it does not offer sufficient resolution to determine the exact functional consequences of these differences. Experimental data, such as mutagenesis studies or higher-resolution structural information, are necessary to clarify how these variations influence substrate binding, active site geometry, and overall enzyme efficiency.

### 2.4. Inhibition of Tg_CA and Selectivity

**Figure 3:**
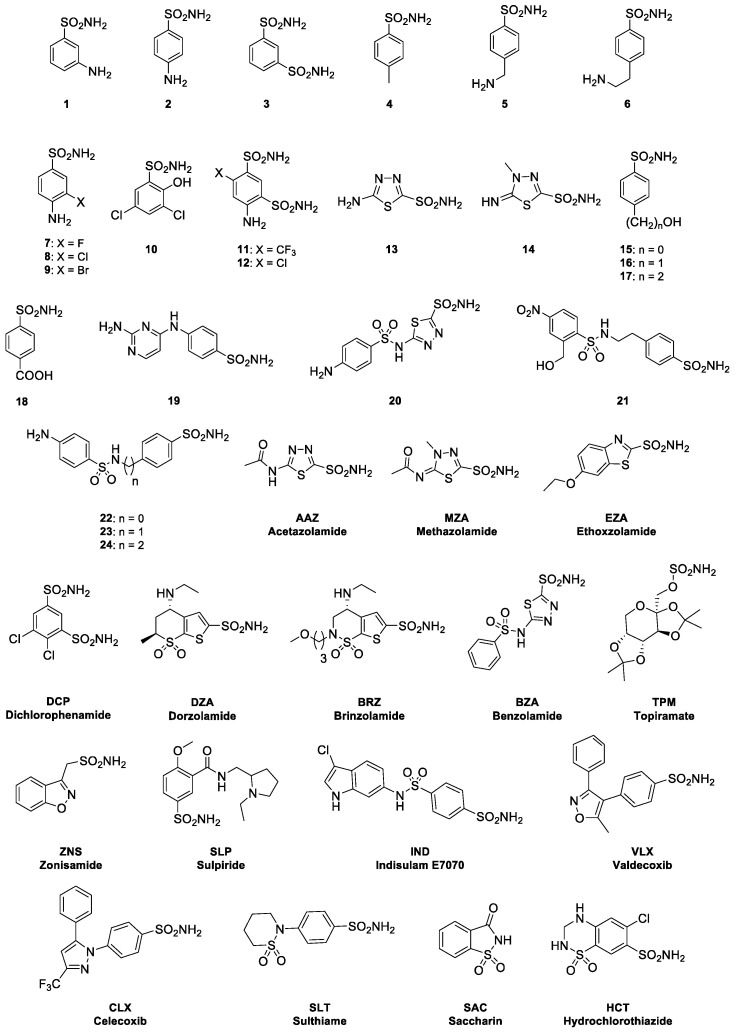
The figure presents the chemical structures of two series of compounds: **1**–**24** and **AAZ**–**HCT**. Compounds **1**–**24**, Experimental sulfonamides and related derivatives, primarily investigated for their ability to inhibit carbonic anhydrases (CAs). **AAZ**–**HCT** series: Acetazolamide (**AAZ**), a prototypical CAI, is commonly used to treat glaucoma by reducing intraocular pressure through the inhibition of aqueous humor production in the ciliary body. **AAZ** is also prescribed for altitude sickness, epilepsy, and periodic paralysis; Methazolamide (**MZA**), a derivative of **AAZ** with enhanced lipid solubility, improving tissue penetration and prolonging its half-life, offering similar therapeutic benefits in glaucoma and related disorders. Ethoxzolamide (**EZA**), primarily used experimentally and occasionally in glaucoma and other CA-related conditions. Dichlorphenamide (**DCP**), primarily used in managing periodic paralysis, though less common for glaucoma, it is crucial in treating specific neuromuscular disorders; Brinzolamide (**BRZ**), a second-generation sulfonamide-based CAI, specifically designed for topical ocular use to lower intraocular pressure in open-angle glaucoma or ocular hypertension; Benzolamide (**BZA**), investigated for glaucoma and diuretic effects but less commonly used today; Zonisamide (**ZNS**), an anticonvulsant primarily used for epilepsy and occasionally for migraines; Sulthiame (**SLP**), used to treat childhood epilepsy syndromes such as Rolandic epilepsy; Indisulam (**IND**), an experimental anticancer drug under investigation for its potential therapeutic effects in oncology; Valdecoxib (**VLX**) and Celecoxib (**CLX**), COX-2 inhibitors used for managing pain and inflammation in conditions such as arthritis; both were withdrawn due to cardiovascular side effects; Sulpiride (**SLT**), an antipsychotic used for managing conditions like schizophrenia and depression; Saccharin (**SAC**), a sulfonamide derivative used primarily as a sweetener rather than a therapeutic agent, though it demonstrates CA inhibition; Hydrochlorothiazide (**HCT**), a diuretic used for managing hypertension, edema, and heart failure. Topiramate (**TPM**), a sulfamate-substituted derivative of a monosaccharide, setting it apart from the classical sulfonamide-based inhibitors of this group. Its structure incorporates a sulfamate group (-SO_2_NH_2_), which is crucial for its biological activity (it is widely used for epilepsy, migraine prophylaxis, and occasionally for bipolar disorder).

Inhibition of carbonic anhydrase (CA) has gained significant attention because of its role in various physiological processes and its implications in diseases such as toxoplasmosis. This study focused on two distinct series of compounds: sulfonamide derivatives (**1**–**24**) and clinically used drugs (**AAZ**–**HCT**) (Figure 3).

Both series represent a collection of small molecules characterized by the presence of a sulfonamide functional group (-SO_2_NH_2_), which is a hallmark of these CAIs. Sulfonamides are particularly effective in targeting the zinc-containing active site of CA by coordinating with the metal ions and disrupting CA activity. Table 2 shows the inhibition constants (K_I_) of these compounds against the two human isoforms (CA I and hCA II) and the *T. gondii* enzyme (Tg_CA), offering insights into their potency and selectivity.

Compounds **1**–**24** and the **AAZ**–**HCT** series exhibited a broad spectrum of inhibition against *T. gondii* CA (Tg_CA), with K_I_ ranging from 17.8 nM to 8450 nM. Compounds **1**, **2**, **3**, **4**, **5**, **6**, **7**, **8**, **9**, **10**, **17**, and **24**, along with **TPM**, **ZNS**, **VLX**, **CLX**, **SLT**, and **HCT**, displayed moderate inhibition of Tg_CA (K_I_ values ranging from 133.8 to 912.4 nM). Among them, for example, compounds **1** (K_I_ = 455.8 nM), **4** (K_I_ = 585.8 nM), and **9** (K_I_ = 344.0 nM) although not the most potent, showed moderate activity; however, they may have favorable pharmacokinetic properties, resulting in promising candidates for development. We want to stress the concept that the therapeutic efficacy of a compound is not solely determined by its potency but also by how it behaves in the body. Compounds **11**, **12**, **13**, **14**, **15**, **16**, **19**, **21**, **22**, **23**, and several clinically used inhibitors, including **AAZ**, **MZA**, **EZA**, **DCP**, **DZA**, **BRZ**, and **IND**, have emerged as highly potent inhibitors of Tg_CA, with K_I_ values ranging from 17.8 to 95.3 nM. However, their inhibition of human isoforms, particularly CA I and CA II, raises concerns regarding selectivity. For instance, compound **20**, with a K_I_ of 17.8 nM for Tg_CA, inhibited human CA I (K_I_ = 6.0 nM) and CA II (K_I_ = 2.0 nM). To enhance selectivity, structural modifications targeting the regions responsible for non-selective inhibition could be explored. Modifying functional groups or side-chains to reduce the binding affinity for human CAs while maintaining potency against Tg_CA is a promising strategy. Clinically used CAIs, such as AAZ, are commonly employed for conditions like glaucoma and epilepsy. Despite good inhibition of human isoforms (K_I_ values of 250 nM for CA I and 12.0 nM for CA II), **AAZ** demonstrates significant activity against Tg_CA (K_I_ = 45.7 nM), making it a candidate for repurposing in toxoplasmosis treatment. However, the dual inhibition of both human and parasitic enzymes necessitates careful evaluation. Compounds such as **MZA**, which exhibit potent inhibition of both human CAs and Tg_CA, could be explored further in cases where human CA inhibition is less of a concern. Compounds such as **DCP**, used to treat periodic paralysis, exhibited stronger inhibition against Tg_CA (K_I_ = 77.5 nM) than against human CA I, suggesting that they could offer better selectivity for the parasite enzyme, although modifications may be required to reduce the inhibition of human CA II. However, **BRZ** may have limited potential for selective Tg_CA inhibition because of its strong effects on both human CAs.

Thus, although some inhibitors show promising activity against Tg_CA, optimizing selectivity remains crucial for minimizing off-target effects. Continued structural and biochemical studies are essential for improving in vitro selectivity and maximizing in vivo therapeutic efficacy against *T. gondii*.

## 3. Materials and Methods

### 3.1. Chemicals and Instruments

The materials and instruments used in this study were sourced from various sources. Isopropyl β-D-1-thiogalactopyranoside (IPTG) and antibiotics were procured from Merck (Darmstadt, Germany). His-Trap FF affinity column and molecular weight markers were supplied by Cytiva (Uppsala, Sweden). Other equipment included the AKTA Prime purification system (Cytiva), SX20 Stopped-Flow instrument (Applied Photophysics, Leatherhead, UK), Thermo Fisher iBright 1500 Imaging System (Thermo Scientific, Waltham, MA, USA), and SDS-PAGE apparatus (Bio-RAD, Hercules, CA, USA). Unless otherwise specified, all the additional reagents were of analytical grade.

### 3.2. Gene Identification, Synthesis, Cloning, and Heterologous Expression

The identification, synthesis, cloning, and expression of the *Toxoplasma gondii* CA (Tg_CA) gene were previously accomplished using a combination of bioinformatics and molecular biology techniques, as detailed in our earlier work [24]. Briefly, the Tg_CA gene was identified using the Protein BLAST program [40] using the query sequences of human α-CAs. A synthetic version of the gene, representing the amino acid region 487–830, was designed and synthesized, then cloned into the pET100/D-TOPO expression vector. The recombinant Tg_CA protein, expressed in *Escherichia coli* BL21 (DE3) codon-plus cells [41], was purified using a nickel-based His-Trap FF affinity column [42], yielding approximately 90% purity. Protein concentration was measured via the Bradford method, and purity was confirmed by 12% SDS-PAGE with Coomassie Brilliant Blue-R staining. Enzymatic activity was evaluated using a CA activity assay, which monitored pH changes during the conversion of CO_2_ to bicarbonate at 0 °C, quantified in Wilbur–Anderson units. For detailed methods, please refer to reference [24]. To assess protein purity, 12% SDS-PAGE with Coomassie Brilliant Blue-R staining was performed [43]. Enzyme activity was evaluated at 0 °C using a CA activity assay based on pH changes during CO_2_ to bicarbonate conversion, with bromothymol blue as an indicator, and quantified in Wilbur–Anderson units [44].

### 3.3. His-Tag Detection of the Fusion Recombinant Protein Tg_CA

His-tag Western blotting was performed using the Pierce Fast Western Blot Kit (Thermo Scientific, Waltham, MA, USA). After protein transfer, the membrane was incubated in Fast Western 1 Wash Buffer to remove the transfer buffer. The membrane was then treated with the primary antibody, diluted to the working concentration, and incubated for 30 min at room temperature (RT) with shaking. Following primary antibody incubation, the membrane was washed twice with 20 mL of Fast Western 1 Wash Buffer. Subsequently, the membrane was incubated for 10 min with the Fast Western Optimized HRP Reagent, diluted to the working concentration, to allow the secondary antibody to be conjugated with horseradish peroxidase (HRP). Finally, the membrane was washed again with Fast Western 1 Wash Buffer to remove any unbound reagents before detection. The blot was developed using the iBright 1500 Imaging System (Thermo Scientific, Waltham, MA, USA), a high-performance imaging platform designed for chemiluminescent and fluorescent detection. The system offers precise, high-resolution imaging with advanced features, such as automatic exposure and quantitative analysis, enabling clear visualization of the chemiluminescent signal generated by the secondary antibody.

### 3.4. Kinetic Constants and Sulfonamide Inhibition Profile of Tg_CA

The kinetic parameters of Tg_CA-catalyzed CO_2_ hydration were measured using an Applied Photophysics stopped-flow spectrophotometer [45]. Phenol red, at a concentration of 0.2 mM, served as the pH indicator, and measurements were performed at an absorbance wavelength of 557 nm. The assay was conducted in 20 mM HEPES buffer (pH 7.5) containing 20 mM NaClO_4_ for human CA II and 20 mM NaCl for Tg_CA to account for the inhibitory effects of perchlorate on the parasite enzyme. Reaction rates were monitored over 10–100 s, with CO_2_ concentrations ranging from 1.7 to 17 mM [46]. Kinetic parameters were derived from Lineweaver–Burk plots, and inhibition constants (K_I_) were calculated using the Cheng–Prusoff equation [47]. Stock solutions of the inhibitors (10–100 mM) were prepared in distilled deionized water and diluted as needed with the assay buffer. Enzyme-inhibitor mixtures were pre-incubated at room temperature for 15 min to ensure complex formation and account for potential inhibitor hydrolysis. The initial reaction rates were determined from the first 5–10% of the reaction data, and the uncatalyzed reaction rates were subtracted to calculate the enzyme-catalyzed rates. Non-linear regression analysis in PRISM 3 software was used to determine the kinetic and inhibition parameters, with each result representing the average of at least three independent experiments [24,47]. Recombinant human CA isoforms were expressed and purified in-house, while salts and small molecules of the highest commercial purity were obtained from Merck (Darmstadt, Germany).

## 4. Conclusions

This study provides a comprehensive characterization of Tg_CA, a zinc-dependent α-CA isolated from *T. gondii*. Bioinformatics analysis confirmed the evolutionary conservation of Tg_CA across species, highlighting its crucial role in maintaining CO_2_/HCO_3_^−^ homeostasis. Recombinant production and purification of Tg_CA yielded a highly homogeneous enzyme, enabling robust kinetic and inhibitory studies. Kinetic analyses revealed that Tg_CA exhibits moderate catalytic efficiency compared to the human isoforms, reflecting adaptations to the unique metabolic demands of the parasite. Structural modeling identified distinct insertions in Tg_CA, suggesting functional specialization, while underscoring the need for high-resolution studies to clarify their impact on enzymatic activity. Additionally, the diverse inhibitory profiles of sulfonamide derivatives (**1**–**24**) and clinically used drugs (**AAZ**–**HCT**) against Tg_CA and human isoforms (hCA I and II) provided critical insights into their potential for treating toxoplasmosis. Compounds **1**, **2**, **3**, **4**, **5**, **6**, **7**, **8**, **9**, **10**, **17**, **24**, along with **TPM**, **ZNS**, **VLX**, **CLX**, **SLT**, and **HCT**, demonstrated moderate potency; others, including **11**, **12**, **13**, **14**, **15**, **16**, **19**, **21**, **22**, and **23**, and several clinically used inhibitors, including **AAZ**, **MZA**, **EZA**, **DCP**, **DZA**, **BRZ**, and **IND**, exhibited exceptional potency. However, their significant activity against human CA isoforms underscores the urgent need for improved selectivity to mitigate the off-target effects. These findings emphasize the importance of targeted structural modifications of these sulfonamide inhibitors to enhance the specificity against Tg_CA. Indeed, there are several examples where modifications to **AAZ**-based scaffolds have demonstrated increased antimicrobial activity. One notable study focusing on *Neisseria gonorrhoeae*, a significant public health concern, investigated modifications to **AAZ** aimed at improving its efficacy against the bacterium’s essential carbonic anhydrase (NgCA) [48]. Through structure–activity relationship (SAR) studies, the authors identified modified **AAZ** analogues (molecules 20 and 23) that exhibited remarkable improvements in potency. These analogues showed minimum inhibitory concentration (MIC) values as low as 0.25 μg/mL—an 8- to 16-fold enhancement in activity compared to **AAZ** [48]. Thus, fine-tuning functional groups, scaffolds, or side-chains holds promise for achieving this balance, thereby reducing the unintended inhibition of human enzymes. Notably, the potential repurposing of clinically established inhibitors such as **AAZ** and **MZA** offers a cost-effective pathway for drug development but requires thorough evaluation to ensure therapeutic safety and efficacy.

The work underscores a dual strategy for advancing Tg_CA inhibitors: (1) repurposing clinically established inhibitors to enable cost-effective therapeutic development, and (2) employing rational design to enhance selectivity. By addressing these challenges, this study lays the foundation for novel targeted treatments for toxoplasmosis, representing a significant step forward in the development of selective and clinically viable enzyme inhibitors.

## Figures and Tables

**Table 1:** Bioinformatic analysis of CA domains identified in *T. gondii* ME49. The table presents a detailed bioinformatic analysis of the carbonic anhydrase (CA) domains identified in the archetypal strain *T. gondii* ME49. This analysis highlighted specific domain hits, their accession codes, descriptions, corresponding amino acid intervals within the protein sequence, and their respective E-values. Legend: Name, the domain or subgroup name identified; Accession, unique identifier for the domain or family from the respective bioinformatics databases; Description, a brief functional characterization of the domain or subgroup; Interval, the range of amino acid residues within the protein sequence where the domain was detected; E-value, statistical significance of the match. Smaller E-values indicate higher confidence in the domain identification.

Name	Accession	Description	Interval	E-Value
Cah	COG3338	Carbonic anhydrase [Inorganic ion transport and metabolism]	607–835	1.35 × 10^−29^
alpha_CA_prokaryotic_like	cd03124	Carbonic anhydrase alpha, prokaryotic-like subfamily	493–835	2.30 × 10^−29^
alpha_CA	cd00326	Carbonic anhydrase alpha (vertebrate-like) group	608–835	2.13 × 10^−26^
Carb_anhydrase	smart01057	Eukaryotic-type carbonic anhydrase	487–836	4.04 × 10^−25^
Carb_anhydrase	pfam00194	Eukaryotic-type carbonic anhydrase	494–835	8.50 × 10^−22^
alpha_CA_I_II_III_XIII	cd03119	Carbonic anhydrase alpha, isozymes I, II, and III and XIII	611–840	1.29 × 10^−14^
alpha_CA_VI_IX_XII_XIV	cd03123	Carbonic anhydrase alpha, isozymes VI, IX, XII and XIV	605–835	1.65 × 10^−13^
alpha_CA_IV_XV_like	cd03117	Carbonic anhydrase alpha, CA_IV, CA_XV, like isozymes	716–835	2.59 × 10^−12^
alpha_CA_VI	cd03125	Carbonic anhydrase alpha, isozyme VI	502–835	9.72 × 10^−12^
alpha_CARP_receptor_like	cd03122	Carbonic anhydrase alpha related protein, receptor_like subfamily	765–844	3.27 × 10^−11^
alpha_CA_XII_XIV	cd03126	Carbonic anhydrase alpha, isozymes XII and XIV	764–835	1.69 × 10^−9^
PLN02179	PLN02179	Carbonic anhydrase	611–807	2.66 × 10^−8^
alpha_CA_VII	cd03149	Carbonic anhydrase alpha, CA isozyme VII_like subgroup	773–835	5.29 × 10^−8^
alpha_CA_IX	cd03150	Carbonic anhydrase alpha, isozyme IX	757–835	6.56 × 10^−8^
PLN02202	PLN02202	Carbonate dehydratase	607–841	1.63 × 10^−7^
alpha_CA_V	cd03118	Carbonic anhydrase alpha, CA isozyme V_like subgroup	773–835	1.93 × 10^−7^
alpha_CARP_VIII	cd03120	Carbonic anhydrase alpha related protein, group VIII	765–819	1.49 × 10^−5^
alpha_CARP_X_XI_like	cd03121	Carbonic anhydrase alpha related protein: groups X, XI and related proteins	781–835	6.52 × 10^−4^

**Table 2:** Inhibition of human isoforms hCA I and hCA II and the enzyme from *T. gondii* α-CA (Tg_CA) with sulfonamides **1**–**24** and the clinically used drugs **AAZ**–**HCT**, using a CO_2_ hydrase stopped-flow assay.

Name	K_I_ (nM) ^a^		
	CA I	CA II	Tg_CA
**1**	28,000	300	455.8
**2**	25,000	240	404.6
**3**	79.0	8.0	447.6
**4**	78,500	320	585.8
**5**	25,000	170	739.5
**6**	21,000	160	793.3
**7**	8300	60.0	168.2
**8**	9800	110	249.2
**9**	6500	40.0	344.0
**10**	7300	54.0	578.2
**11**	5800	63.0	65.1
**12**	8400	75.0	73.8
**13**	8600	60.0	57.5
**14**	9300	19.0	61.0
**15**	5500	80.0	76.4
**16**	9500	94.0	91.6
**17**	21,000	125	262.5
**18**	164	46.0	85.3
**19**	109	33.0	72.5
**20**	6.0	2.0	17.8
**21**	69.0	11.0	94.7
**22**	164	46.0	91.2
**23**	109	33.0	93.4
**24**	95.0	30.0	204.1
**AAZ**	250	12.0	45.7
**MZA**	50.0	14.0	24.7
**EZA**	25.0	8.0	27.2
**DCP**	1200	38.0	77.5
**DZA**	50,000	9.0	95.3
**BRZ**	45,000	3.0	77.8
**BZA**	15.0	9.0	62.1
**TPM**	250	10.0	720.4
**ZNS**	56.0	35.0	912.4
**SLP**	1200	40.0	1812
**IND**	31.0	15.0	84.0
**VLX**	54,000	43.0	425.9
**CLX**	50,000	21.0	508.2
**SLT**	374	9.0	133.8
**SAC**	18,540	5959	8450
**HCT**	328	290	839.2

^a^ Mean from 3 different assays, by a stopped-flow technique (errors were in the range of ±5–10% of the reported values).

## Data Availability

The original contributions presented in the study are included in the article, further inquiries can be directed to the corresponding author(s).
